# Chk-mate on resistance to kinase inhibitors

**DOI:** 10.18632/oncotarget.25802

**Published:** 2018-08-03

**Authors:** Paul Shapiro

**Affiliations:** Department of Pharmaceutical Sciences, University of Maryland, Baltimore, MD, USA

**Keywords:** melanoma, kinase inhibitors, drug resistance

The invariable development of drug resistance represents a major obstacle to the successful treatment of cancer with kinase inhibitors. In a recent study published in *Oncotarget*, Hwang *et al.* have demonstrated that inhibitors of cell cycle checkpoint kinases can restore sensitivity of melanoma cells that have acquired resistance to BRaf kinase inhibitors. Approximately 50% of melanoma tumors contain activating mutations in BRaf, which promotes cancer cell proliferation and survival through constitutive activation of the extracellular signal-regulated kinase-1 and 2 (ERK1/2) proteins. The dependence of these melanomas on mutated BRaf validated the development of oncogenic-selective BRaf inhibitors [1]. However, despite very promising initial responses, patients rapidly develop resistance to BRaf inhibitors often through mechanisms that re-activate the ERK1/2 proteins [2]. The utility of targeted inhibition of both BRaf and cell cycle checkpoint kinases in the treatment of melanoma has not been thoroughly evaluated.

The cell cycle checkpoint kinases-1 and 2 (Chk1/2) are activated in response to DNA damage and promote cell survival by initiating cell cycle arrest to allow for DNA repair. A wealth of preclinical data has indicated that blocking Chk1/2 activity may be an effective approach to enhance DNA damage and ultimately death of cancer cells. These finding have supported the development of Chk1 selective inhibitors, including PF477736 [3], used in the studies by Hwang *et al*., and LY2606368 [4], which is being evaluated in several clinical trials to treat cancer including squamous cell carcinomas [5] and ovarian cancer that is refractory or resistant to the DNA damage induced by cisplatin [6]. A limitation of using Chk inhibitors as monotherapy is the observed compensatory activation of ERK1/2 and other proteins that promote cancer cell survival, which supports the idea that combining Chk and ERK1/2 inhibitors would achieve greater clinical benefits [7]. Indeed, previous studies showed that a combination of Chk2 and ERK1/2 inhibition is superior for killing diffuse large B-cell lymphoma cells compared to inhibiting each protein individually [8].

Based on clinical data showing that melanoma patients with low levels of Chk1 expression had better survival outcomes compared to patients with high Chk1 expression, Hwang *et al.* tested whether the Chk1 inhibitor PF477736 was effective in melanoma cells that were sensitive or resistant to BRaf inhibitors. Interestingly, some drug resistant cell lines were 3 times more sensitive to PF477736 than the drug sensitive cells indicating that cellular changes that confer resistance to BRaf inhibitors are highly dependent on Chk1 activity. The authors went on to show that Chk1 inhibition can restore the sensitivity of BRaf inhibitor resistant melanoma cells to the killing effects of the clinically relevant BRaf inhibitor, PLX-4032, also known by its generic drug name as vemurafenib. While the specific mechanisms responsible for Chk1 inhibitors restoration of sensitivity to BRaf inhibitors in the drug resistant cells remains to be determined, the authors provide evidence that combining PLX4032 and PF477736 alters the phosphorylation pattern of Chk1 that may result in Chk1 protein degradation and changes in intracellular location. Further studies are needed to clarify the molecular changes induced by Chk1 inhibitors that facilitate the restoration of BRaf inhibitor sensitivity in drug resistant melanoma cells. Nonetheless, the current studies by Hwang *et al.* provide a compelling rationale to evaluate combining Chk1 and BRaf inhibitor therapies in the treatment of melanomas containing mutant BRaf and placing a check on the induction of drug resistance.
